# Association of Midlife Cardiovascular Risk Profiles With Cerebral Perfusion at Older Ages

**DOI:** 10.1001/jamanetworkopen.2019.5776

**Published:** 2019-06-21

**Authors:** Sana Suri, Anya Topiwala, Michael A. Chappell, Thomas W. Okell, Enikő Zsoldos, Archana Singh-Manoux, Mika Kivimäki, Clare E. Mackay, Klaus P. Ebmeier

**Affiliations:** 1 Department of Psychiatry, Warneford Hospital, University of Oxford, Oxford, United Kingdom; 2 Wellcome Centre for Integrative Neuroimaging, University of Oxford, Oxford, United Kingdom; 3 Institute of Biomedical Engineering, University of Oxford, Oxford, United Kingdom; 4 Inserm U1153, Epidemiology of Ageing and Neurodegenerative Diseases, Université Paris Descartes, Paris, France; 5 Department of Epidemiology and Public Health, University College London, London, United Kingdom

## Abstract

**Importance:**

Poor cardiovascular health is an established risk factor for dementia, but little is known about its association with brain physiology in older adults.

**Objective:**

To examine the association of cardiovascular risk factors, measured repeatedly during a 20-year period, with cerebral perfusion at older ages.

**Design, Setting, and Participants:**

In this longitudinal cohort study, individuals were selected from the Whitehall II Imaging Substudy. Participants were included if they had no clinical diagnosis of dementia, had no gross brain structural abnormalities on magnetic resonance imaging scans, and had received pseudocontinuous arterial spin labeling magnetic resonance imaging. Cardiovascular risk was measured at 5-year intervals across 5 phases from September 1991 to October 2013. Arterial spin labeling scans were acquired between April 2014 and December 2014. Data analysis was performed from June 2016 to September 2018.

**Exposures:**

Framingham Risk Score (FRS) for cardiovascular disease, comprising age, sex, high-density lipoprotein cholesterol level, total cholesterol level, systolic blood pressure, use of antihypertensive medications, cigarette smoking, and diabetes, was assessed at 5 visits.

**Main Outcomes and Measures:**

Cerebral blood flow (CBF; in milliliters per 100 g of tissue per minute) was quantified with pseudocontinuous arterial spin labeling magnetic resonance imaging.

**Results:**

Of 116 adult participants, 99 (85.3%) were men. At the first examination, mean (SD) age was 47.1 (5.0) years; at the last examination, mean (SD) age was 67.4 (4.9) years. Mean (SD) age at MRI scan was 69.3 (5.0) years. Log-FRS increased with time (B = 0.058; 95% CI, 0.044 to 0.072; *P* < .001). Higher cumulative FRS over the 20-year period (measured as the integral of the rate of change of log-FRS) was associated with lower gray matter CBF (B = −0.513; 95% CI −0.802 to −0.224; *P* < .001) after adjustment for age, sex, education, socioeconomic status, cognitive status, arterial transit time, use of statins, and weekly alcohol consumption. Voxelwise analyses revealed that this association was significant in 39.6% of gray matter regions, including the posterior cingulate, precuneus, lateral parietal cortex, occipital cortex, hippocampi, and parahippocampal gyrus. The strength of the association of higher log-FRS with lower CBF decreased progressively from the first examination (*R*^2^ = 0.253; B = −10.816; 99% CI −18.375 to −3.257; *P* < .001) to the last (*R*^2^ = 0.188; B = −7.139; 99% CI −14.861 to 0.582; *P* = .02), such that the most recent FRS measurement at mean (SD) age 67.4 (4.9) years was not significantly associated with CBF with a Bonferroni-corrected *P* < .01 .

**Conclusions and Relevance:**

Cardiovascular risk in midlife was significantly associated with lower gray matter perfusion at older ages, but this association was not significant for cardiovascular risk in later life. This finding could inform the timing of cardiovascular interventions so as to be optimally effective.

## Key Points

**Question:**

Is midlife cardiovascular risk associated with cerebral blood flow in older age, and does this association vary with age?

**Findings:**

In this longitudinal cohort study of 116 older adults without dementia, higher cardiovascular risk scores during a 20-year period were significantly associated with lower cerebral blood flow to the medial temporal, parietal, and occipital cortices. The association varied during the life span such that cardiovascular risk in midlife but not in later life was significantly associated with cerebral hypoperfusion in older age.

**Meaning:**

Because cerebral hypoperfusion is an early mechanism in Alzheimer disease and vascular dementia, these findings may inform the development of dementia prevention strategies aimed at managing cardiovascular health.

## Teaser

This longitudinal cohort study examines the association of midlife cardiovascular risk factors with cerebral perfusion at older ages among adults without dementia.

## Introduction

Dementia and cardiovascular diseases (CVDs) share many risk factors, including hypertension, smoking, type 2 diabetes, hypercholesterolemia, and obesity.^1^ These cardiovascular risk factors are potentially modifiable, and their timely management could help prevent nearly one-third of dementia cases worldwide.^2,3^ In epidemiological studies, cardiovascular risk in midlife rather than old age has been associated with increased dementia risk. For example, obesity in midlife (ages 40-60 years) is associated with an elevated risk of dementia,^4,5^ but owing to loss of weight during the preclinical stage of dementia, short-term follow-up studies of people older than 70 years have reported reduced dementia incidence in individuals with obesity.^6,7,8^ The association of dementia prevalence with hypertension may also be age dependent, with evidence of positive associations with midlife hypertension but no association or inverse associations with later-life hypertension.^9,10^

The brain physiology underlying these associations remains unclear, and in this study, we examined whether cerebral blood flow (CBF) may play a role. Reduced CBF is an important biological mechanism in dementia, and hypoperfusion in patients with mild cognitive impairment, vascular dementia, and Alzheimer disease has been well documented.^11,12,13,14,15,16^ A 2016 Alzheimer Disease Neuroimaging Initiative study^17^ found that of several disease biomarkers, such as brain atrophy, metabolism, CBF, functional connectivity, β-amyloid deposition, plasma and cerebrospinal fluid markers, and cognition, the earliest pathological event in Alzheimer progression was reduced CBF. Cerebrovascular dysregulation may therefore precede and even accelerate neurodegeneration, and CBF in particular has emerged as a promising imaging biomarker for preventive interventions.^13,18,19,20^

We examined whether cardiovascular risk measured repeatedly during a 20-year period in midlife was associated with CBF in later life, with the aim of identifying the age at which an association was strongest. We operationalized vascular risk as the Framingham Risk Score (FRS) for CVD, a widely cited score that combines multiple risk factors to estimate 10-year risk of general CVD.^21^ The FRS has been thoroughly validated for assessing cardiac health in primary care and is also a reliable predictor of cognitive decline,^22^ cerebrovascular lesions, such as white matter hyperintensities,^23^ and progression to dementia.^24^ We quantified CBF noninvasively with magnetic resonance imaging (MRI)–based pseudocontinuous arterial spin labeling (pCASL)^25^ and hypothesized that higher FRS would be associated with reduced CBF. We did not make any prior assumptions about affected brain areas and instead performed voxelwise analyses of the whole brain.

## Methods

### Design, Setting, and Participants

Data were drawn from the Whitehall II Imaging Substudy, a cohort of 800 UK civil servants aged 60 to 85 years who received multimodal brain MRI scans at the Wellcome Centre for Integrative Neuroimaging, University of Oxford, Oxford, United Kingdom, between April 2012 and December 2016. From April 2014 to December 2014, pCASL MRIs were conducted in a subset of this cohort (145 participants), and only participants who received a pCASL scan were included in this study. Whitehall II Imaging Substudy participants were randomly selected from the parent Whitehall II Study, an ongoing prospective cohort study established in 1985 at University College London, London, United Kingdom. The parent cohort included 10 308 volunteers aged 35 to 55 years at the time who have been observed for more than 30 years in 12 phases. Cardiovascular risk was measured at 5 phases: phase 3 (1991-1994), phase 5 (1997-1999), phase 7 (2002-2004), phase 9 (2007-2009), and phase 11 (2011-2013). Participants have shown a mean response rate of 77.14% from phase 1 to phase 11. Detailed protocols for the Whitehall II Study and Imaging Substudy have been described previously.^26,27^

Of the 145 Whitehall II Imaging Substudy participants with pCASL scans, we excluded those with gross structural MRI abnormalities (eg, large strokes, tumors, and cysts; 7 participants [4.8%]), missing FRS data at phases 3 and 11 (19 participants [13.1%]), and missing FRS data from 3 or more phases (3 participants [2.1%]). Accordingly, data from 116 participants (80.0%) were analyzed in this study, and none had a clinical diagnosis of dementia at the time of the MRI scan. All participants provided written informed consent, and the study was approved by the University of Oxford Medical Sciences Interdivisional Research Ethics Committee as part of the larger study. This report follows the Strengthening the Reporting of Observational Studies in Epidemiology (STROBE) reporting guideline.^28^

### Vascular Risk

The primary exposure variable, FRS, was measured at 5 Whitehall II Study phases using information on age, sex, high-density lipoprotein cholesterol level, total cholesterol level, systolic blood pressure, use of antihypertensive medication, cigarette smoking, and type 2 diabetes (eAppendix in the Supplement).^29,30^ At each phase, FRS was only computed for participants who did not have prevalent CVD at that phase. For example, if a participant had prevalent CVD at phases 5 and 7, FRS was only computed for phases 3, 9, and 11. Cardiovascular disease was defined as having myocardial infarction, angina, or stroke diagnosed with clinical examination, electrocardiography, and medical records.^30^ Raw scores were converted to 10-year risk or predicted probability of incident CVD expressed as a percentage.^21^ Risk of CVD is typically categorized as low if FRS is less than or equal to 10%, moderate if FRS is between 10% and 20%, and high if FRS is greater than or equal to 20%. In the Whitehall II Substudy cohort, FRS reliably predicted CVD^31^ and cognitive decline.^22,29^

### Cerebral Blood Flow

The primary outcome was CBF, quantified as the rate of delivery of arterial blood to brain tissue (milliliters of blood per 100 g of tissue per minute) using pCASL MRI.^25^ Magnetic resonance imaging scans were acquired on a 3-T Magnetom Verio Scanner with a 32-channel head coil (Siemens). A multiple postlabeling delay pCASL scan was used to quantify absolute resting CBF and arterial transit time (repetition time, 4240 milliseconds; echo time, 13 milliseconds; voxel size, 3.4 × 3.4 × 4.5 mm; flip angle, 90°; slice thickness, 4.5 mm; labeling duration, 1400 milliseconds; postlabel delays, 0.25, 0.50, 0.75, 1.0, 1.25, 1.5, and 1.75 seconds).^32^ Two calibration scans (repetition time, 10 000 milliseconds; echo time, 13 milliseconds) were acquired to calibrate the pCASL perfusion-weighted signal via the equilibrium magnetization of blood. T1-weighted multiecho magnetization-prepared rapid gradient echo structural MRI scans (voxel size, 1 mm^3^; repetition time, 2530 milliseconds; echo times, 1.79, 3.65, 5.51, and 7.37 milliseconds) were used for registration and partial-volume correction of the perfusion data. Images were processed using FMRIB Software Library tools version 6.0 (FSL).^33^ Absolute resting perfusion maps were generated using the Bayesian Inference for Arterial Spin Labeling (BASIL) MRI tool in FSL, which uses a variational Bayes approach to perform a nonlinear fit of the general kinetic model to the pCASL data for all voxels in the brain.^34^ Pseudocontinuous arterial spin labeling scans were preprocessed, partial-volume corrected, and registered to MNI 152 standard space (Montreal Neurological Institute) to obtain gray matter (GM) CBF maps (eAppendix in the Supplement).^32,35,36,37^

### Statistical Analysis

#### Linear Mixed-Effects Model

We examined the association of CBF with (1) cumulative FRS from phases 3 to 11, (2) longitudinal change in FRS, and (3) FRS at each phase. Phase 1 (1985-1988) of the Whitehall II Study corresponded to time 0, and FRS was tracked for approximately 20 years from phase 3 (1991-1994) to phase 11 (2011-2013). Longitudinal change in FRS was calculated using linear mixed-effects models with maximum likelihood estimation in R version 1.1.463 (The R Foundation) using the nlme package. The model implemented a continuous autoregressive moving-average correlation structure to consider correlations between repeated measures on the same individual. Both the intercepts and slope (time) were fitted as random effects. Adding a quadratic term for time as a fixed effect significantly improved the model fit (χ^2^_1_ = 4.2; deviance, 394; *P* = .04). Framingham Risk Scores were logarithmically transformed owing to the positively skewed distribution of the standardized residuals. Cumulative FRS during the 20-year period was estimated as the integral of the rate of change of log-FRS (area under the y-curve) calculated with trapezoidal integration. For all participants, (1) cumulative 20-year FRS, (2) intercepts (ie, predicted FRS at phase 1), and (3) slopes (ie, rate of change of FRS from phases 3-11) of the mixed-effects model were extracted for subsequent analyses.

#### MRI Analyses

Voxelwise general linear modeling was performed in FSL. Participants’ standard-space GM perfusion maps were concatenated into a 4-dimensional file. This was submitted to FSL-Randomize to perform a permutation-based nonparametric test with 5000 permutations. The threshold-free cluster enhancement option was used, and a standard MNI 152 GM mask (threshold, 35) was supplied in FSL-Randomize. In the first voxelwise analysis conducted on 116 participants, cumulative FRS was the independent variable in a covariate-adjusted general linear model, and results were reported at a familywise error–corrected, 1-sided *P* < .05. In the second voxelwise analysis, FRS scores from the 5 phases were entered in 5 separate general linear models, and results were reported at familywise error–corrected, Bonferroni-corrected, 1-sided *P* < .01. The second voxelwise analysis was conducted on 98 participants who had complete FRS data across all 5 phases. As voxelwise statistics are nonparametric, FRS scores were not log-transformed for these analyses.

#### Region of Interest Analyses

Mean global GM CBF was extracted from native-space CBF maps using the MNI 152 GM mask and regressed against (1) cumulative FRS, (2) FRS slopes and intercepts, and (3) log-FRS at each phase. Differences in the contribution of phase 3 vs phase 11 log-FRS to GM CBF were compared using hierarchical regression models. Mean CBF to the frontal, temporal, parietal, and occipital lobes was extracted from native-space perfusion maps using region of interest masks derived from the MNI Structural Atlas (threshold, 40). Separate linear regressions of phase 3 log FRS vs CBF to each lobe were performed, and significance was accepted at a Bonferroni-corrected, 1-sided *P* < .0125 to correct for 4 regressions (1 for each region of interest).

#### Covariates

Covariates reported to influence CBF and cardiovascular risk were included in all models. These included age, sex, education, socioeconomic status, cognitive status, mean arterial transit time for GM, statin medication, and alcohol consumption. At the MRI phase, education was calculated as the total years of full-time and part-time education; cognitive status was assessed using the Montreal Cognitive Assessment^38^; and statin use was classified according to British National Formulary Class 2.12. Alcohol intake was assessed by self-report questionnaires administered at phase 9, phase 11, and the MRI phase. Mean alcohol consumption was calculated as total units per week of alcohol consumed averaged across the 3 phases. Socioeconomic status was defined based on the highest civil service employment grade achieved at phase 3 (highest, grade 1; lowest, grade 4). Secondary associations of covariates with CBF have been reported but not further interpreted.

#### Additional Analyses

As the equation used to derive FRS includes age as a risk factor, we ran further analyses to exclude a biased contribution of age to our results. We reran all models with an additional covariate for quadratic age, and this did not change the results (data not shown). However, because quadratic age introduced high multicollinearity in the models and was not significantly associated with CBF (B = −0.0024; 95% CI, −0.0056 to 0.0009; *P* = .13), the results presented do not include quadratic age as a covariate. We also computed a modified FRS without the age component in the equation, and the results remained consistent (eTable 1 in the Supplement). Further, apolipoprotein E genotype was available for a subset of participants (84 [72.4%]), and correcting for apolipoprotein E in a separate subanalysis did not alter the results (data not shown).

## Results

### Participant Characteristics

The demographic characteristics and FRS profiles of the 116 included participants were not significantly different from the complete Whitehall II Imaging Substudy cohort (eTable 2 in the Supplement). Participant characteristics at the MRI phase are shown in Table 1; 99 (85.3%) were male. At the first examination, mean (SD) age was 47.1 (5.0) years; at the last examination, mean (SD) age was 67.4 (4.9) years. Mean (SD) age at MRI scan was 69.3 (5.0) years. Participants had a mean (SD) duration of education of 15.8 (3.4) years. At phase 3, 86 participants (74.1%) had a low risk of developing CVD in 10 years, 24 (20.7%) had a moderate risk, and 6 (5.2%) had a high risk (Table 2).^21^ Log-transformed FRS increased significantly with time (B, 0.058; 95% CI, 0.044 to 0.072; *t*_437_ = 8.1; *P* < .001).

**Table 1.** Participant Characteristics at the Magnetic Resonance Imaging Phase in 2014

Characteristic	Mean (SD) [Range]^a^
Total participants, No.	116
Demographic characteristics	
Age, y	69.3 (4.96) [61.94-80.94]
Sex, No. (%)	
Men	99 (85.3)
Women	17 (14.7)
Socioeconomic status, No. (%)	
1	13 (11.2)
2	96 (82.8)
3	6 (5.2)
4	1 (0.9)
Education, full-time and half-time, y	15.83 (3.39) [7.50-28.50]
MOCA score, median (IQR) [range]	27 (25-29) [19-30]
Cardiovascular health	
Blood pressure, mm Hg	
Systolic	140.97 (17.02) [106.00-210.00]
Diastolic	75.83 (10.18) [54.00-102.00]
Antihypertensive medication use, No. (%)^b^	37 (31.9)
Statin use, No. (%)	50 (43.1)
Cigarette smokers, No. (%)	5 (4.3)
Type 2 diabetes, No. (%)	8 (6.9)
Alcohol consumption during 3 phases, median (IQR) [range], units/wk	10.13 (4.66-15.92) [0-55.64]
BMI	25.72 (3.79) [14.29-35.77]
*ApoE* E3/E4 or E4/E4 carriers, No./total No. (%)^c^	21/84 (25.0)
Brain volumes, % of TBV	
TBV, mL	1455.95 (128.51) [1172.27-1927.78]
Gray matter volume	36.99 (2.08) [31.05-42.95]
White matter volume	37.22 (1.61) [33.30-43.88]
Cerebrospinal fluid volume	25.79 (2.66) [20.91-32.53]
Cerebral blood flow, mL/100 g/min	
Total gray matter	56.05 (12.21) [31.58-89.44]
Frontal lobe	55.26 (13.70) [26.84-92.20]
Parietal lobe	64.49 (16.28) [27.95-113.37]
Temporal lobe	53.90 (10.98) [27.70-80.07]
Occipital lobe	56.61 (18.23) [19.41-114.00]

Abbreviations: *ApoE*, apolipoprotein E; BMI, body mass index (calculated as weight in kilograms divided by height in meters squared); IQR, interquartile range; MOCA, Montreal Cognitive Assessment; TBV, total brain volume.

^a^ Values are displayed as mean (SD) for normally distributed data, and median (IQR) for data that are not normally distributed. Ranges represent the minimum and maximum value for the data.

^b^ Antihypertensive medications included diuretics, β-blockers, angiotensin-converting enzyme inhibitors, and calcium channel blockers.

^c^ APOE genotype was only available for 84 of 116 participants.

**Table 2.** Participant Characteristics for 5 Phases of Whitehall II Study

Characteristic	Phase 3, 1991-1994	Phase 5, 1997-1999	Phase 7, 2002-2004	Phase 9, 2007-2009	Phase 11, 2011-2013
Total participants	116	106	109	108	116
FRS %, median (IQR) [range]^a^	6.92 (4.65-10.22) [0.88-24.68]	8.99 (5.76-14.84) [1.17-32.33]	12.80 (9.01-18.18) [1.43-41.91]	15.55 (9.63-21.21) [3.06-58.00]	16.39 (11.44-24.59) [2.85-55.08]
FRS, No. (%)^b^					
Low	86 (74.1)	58 (54.7)	36 (33.3)	30 (27.8)	16 (13.8)
Moderate	24 (20.7)	39 (36.8)	52 (47.7)	48 (44.4)	58 (50.0)
High	6 (5.2)	9 (8.5)	21 (19.3)	30 (27.8)	42 (36.2)
Age, mean (SD) [range], y	47.1 (5.0) [40.1-58.9]	52.9 (4.9) [45.7-64.8]	58.5 (5.0) [51.4-70.1]	63.5 (5.0) [56.4-75.3]	67.4 (4.9) [60.5-79.5]

Abbreviations: FRS, Framingham Risk Score; IQR, interquartile range.

^a^ Framingham Risk Score is expressed as a percentage of 10-year risk of cardiovascular disease.

^b^ Low risk defined as FRS less than or equal to 10%; moderate risk, FRS between 10% and 20%; and high risk, FRS greater than or equal to 20%.

### Association of Cumulative FRS With CBF

**Figure 1.**
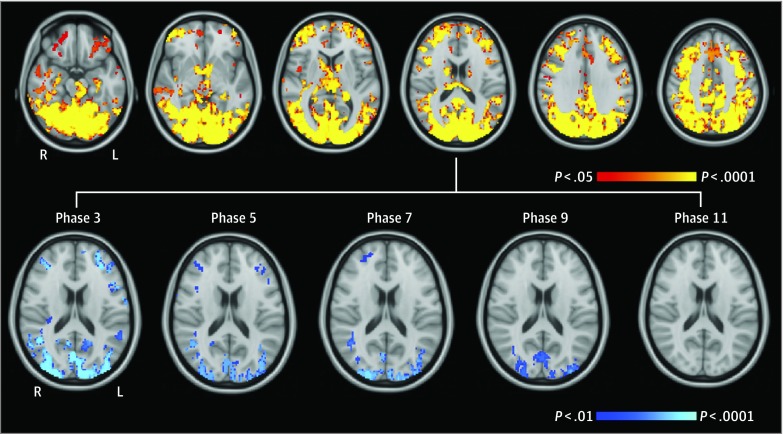
Voxelwise Association of Framingham Risk Scores With Cerebral Blood Flow Top, Red-yellow clusters denote regions showing a significant negative association of cumulative Framingham Risk Score with gray matter cerebral blood flow during 20 years (thresholded at familywise error–corrected *P* < .05). From left to right, horizontal slices are displayed at *z* = −24, *z* = −12, *z* = 0, *z* = 12, *z* = 24, and *z* = 36 in coordinate space (millimeters). Bottom, Blue clusters denote regions showing a significant negative association of Framingham Risk Score with gray matter cerebral blood flow at 5 study phases (thresholded at familywise error–corrected and Bonferroni-corrected *P* < .01). The association of Framingham Risk Score with cerebral blood flow became progressively less widespread from phase 3 to phase 9 and was not statistically significant for phase 11 risk scores. L indicates left; R, right.

Cumulative FRS during 20 years (ie, integrals of the change in log-FRS) significantly explained 11.1% of the variance in GM CBF extracted from perfusion maps (*R*^2^ = 0.111; B = −0.346; 95% CI, −0.528 to −0.165; *P* < .001). Adding covariates to the model explained an additional 10.4% of the variance in GM CBF (*R*^2^ = 0.215; *P *for the model = .002), and cumulative FRS remained significantly associated with CBF (B = −0.513; 95% CI, −0.802 to −0.224: *P* < .001). Voxelwise regression revealed that higher cumulative FRS was significantly associated with lower CBF to 39.6% of GM areas, including the posterior cingulate, precuneus, lateral parietal cortex, occipital cortex, bilateral caudate, thalamus, hippocampus, parahippocampal gyrus, anterior cingulate, middle frontal gyrus, and frontal pole (Figure 1).

We examined the association of CBF with the intercepts and slopes of FRS trajectories. The intercepts alone significantly explained 11.5% of the variance in GM CBF (*R*^2^ = 0.115; B = −6.488; 95% CI, −9.824 to −3.152; *P* < .001), and adding FRS slopes to the model did not significantly change model fit (*F*_1,114_ = 0.01; *P* = .94). In a covariate-adjusted model (*R*^2^ = 0.220; *P *for the model = .002), FRS intercepts but not slopes were significantly associated with CBF (FRS intercepts: B = −10.497; 95% CI, −17.424 to −3.569; *P* = .003; FRS slopes: B = −57.785; 95% CI, −478.573 to 363.004; *P* = .79).

### Association of FRS at Each Phase With CBF

Voxelwise analysis in 98 participants with complete FRS measurements from all phases revealed that the strength of the association of FRS with CBF varied across phases. In covariate-adjusted models, FRS at phase 3 was significantly associated with lower CBF to 16.9% of GM, including the posterior cingulate, precuneus, lateral parietal cortex, middle frontal cortex, and occipital cortex at the familywise error–corrected and Bonferroni-corrected *P* < .01 (Figure 1). However, this association became progressively less widespread over time, with significant associations at phases 5, 7, and 9 localized to 8.9%, 7.2%, and 5.5%, respectively, of GM. The FRS measurement at phase 11 was not significantly associated with CBF. To derive effect sizes, mean CBF was extracted from GM perfusion maps and entered into a linear regression against log-FRS at each phase. Consistent with the voxelwise results, the strength of the association of FRS with CBF decreased from the first examination at phase 3 (*R*^2^ = 0.253; B = −10.816; 99% CI, −18.375 to −3.257; *P* < .001) to phase 5 (*R*^2^ = 0.218; B = −8.288; 99% CI, −15.353 to −1.223; *P* = .003) and phase 7 (*R*^2^ = 0.220; B = −8.511; 99% CI, −15.660 to −1.361; *P* = .002) as well as the later examinations at phase 9 (*R*^2^ = 0.169; B = −5.743; 99% CI, −13.463 to 1.977; *P* = .05) and phase 11 (*R*^2^ = 0.188; B = −7.139; 99% CI, −14.861 to 0.582; *P* = .02). Adding phase 11 log-FRS to the phase 3 model did not significantly change model fit (*F*_1,87_ = 0.39; *P* = .53); however, adding phase 3 log-FRS to the phase 11 model significantly improved model fit (*F*_1,87_ = 8.08; *P* = .006).

**Figure 2.**
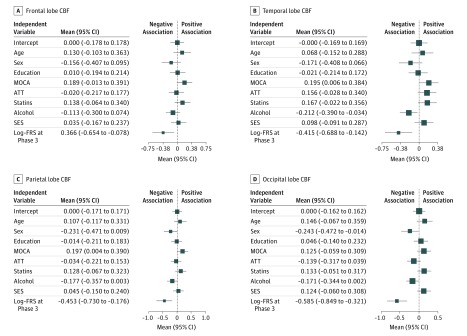
Forest Plot Summarizing Standardized Coefficients for the Regression of Phase 3 Log–Framingham Risk Scores (FRS) With Cerebral Blood Flow (CBF) (N = 116) Mean values denote the standardized regression coefficients for each independent variable. Log-FRS at phase 3 is significantly associated with CBF to the temporal, parietal, and occipital lobes at a Bonferroni-corrected *P* < .0125. ATT indicates arterial transit time; MOCA, Montreal Cognitive Assessment score; and SES, socioeconomic status.

Cerebral blood flow to the frontal, temporal, parietal, and occipital lobes was extracted using region of interest masks. Consistent with the voxelwise results, phase 3 log-FRS was associated with parietal, occipital, and temporal lobe CBF at Bonferroni-corrected *P* < .0125. We also noted secondary associations of covariates with CBF at an uncorrected *P* < .05. Montreal Cognitive Assessment scores were positively associated with CBF to the temporal and parietal lobes, whereas male sex was negatively associated with CBF to the occipital lobe and alcohol consumption was negatively associated with CBF to the temporal lobe (Table 3; Figure 2).

**Table 3.** Association of Phase 3 Framingham Risk Scores (FRS) With Cerebral Blood Flow

Model	Lobe CBF															
	Frontal				Temporal				Parietal				Occipital			
	*R*^2^, %	*P* Value for Model	B (SE)	*P* Value for FRS	*R*^2^, %	*P* Value for Model	B (SE)	*P* Value for FRS	*R*^2^, %	*P* Value for Model	B (SE)	*P* Value for FRS	*R*^2^, %	*P* Value for Model	B (SE)	*P* Value for FRS
Model 1^a^	5.6	.01	−5.118 (1.968)	.01^b^	8.5	.001	−5.063 (1.552)	.001^c^	9.8	.001	−8.060 (2.285)	.001^c^	17.5	<.001	−12.034 (2.448)	<.001^c^
Model 2^d^	12.0	.12	−7.924 (3.188)	.02^b^	21.6	.002^c^	−7.198 (2.411)	.004^c^	19.1	.006	−11.633 (3.634)	.002^c^	26.7	<.001	−16.846 (3.872)	<.001^c^

Abbreviation: CBF, cerebral blood flow.

^a^ Model 1 examined the association of log–Framingham Risk Scores at phase 3 with CBF to the frontal, temporal, parietal, and occipital lobes.

^b^ Associations significant at uncorrected *P* < .05.

^c^ Associations statistically significant at Bonferroni-corrected *P* < .0125.

^d^ Model 2 included additional covariates: age, sex, education, cognitive status, socioeconomic status, arterial transit time, statin medication use, and units per week of alcohol consumed.

## Discussion

The 2-hit vascular hypothesis^20^ and heart-brain^19^ models of dementia propose that cerebral hypoperfusion may be a key biological pathway of the association of cardiovascular risk with dementia. In support of these models, we observed that cumulative cardiovascular risk during a 20-year period in midlife was significantly associated with lower CBF in later life. This association was fairly widespread, covering approximately 39.6% of GM, and it was most prominent in the posterior and parietal cortices. Our longitudinal analyses revealed an age-dependent pattern so that midlife vascular risk profiles rather than later-life vascular risk profiles showed the strongest association with CBF in older age. The earliest risk measurements (phase 3) made a unique and significant contribution to CBF over and above that of the latest risk scores (phase 11) but not vice versa. This emphasizes the importance of an early start for dementia prevention by cardiovascular interventions.

Obesity, hypercholesterolemia, hypertension, and dysfunctional insulin signaling in diabetes are accompanied by increased intima-media thickness, progressive hardening of large arteries, and excess pulsatility, all of which can ultimately affect the brain’s blood supply.^18,39,40,41,42,43,44^ The prefrontal, posterior cingulate, and occipital cortices are particularly susceptible to vascular damage,^45^ and this is consistent with our observations of lower CBF in these areas.

Kaffashian et al^29^ have previously reported that higher midlife FRS predicts faster 10-year cognitive decline in more than 5000 Whitehall II Study participants. Our study was conducted in a randomly selected subsample of 116 adults from this cohort and suggests that cerebral hypoperfusion may contribute to this association. The role of reduced CBF in the pathogenesis of vascular and Alzheimer dementia has been extensively reviewed.^12,18,20,46^ Chronic hypoperfusion can damage the blood-brain barrier. This eventually reduces the clearance of toxic β-amyloid and increases the leakage of inflammatory markers into the central nervous system, causing downstream metabolic and inflammatory dysfunction.^12,42,46^ Severe hypoperfusion accelerates the accumulation of white matter hyperintensities, reactive oxygen species, and amyloid and hyperphosphorylated tau deposits.^47,48,49^ Reduced CBF is also associated with the severity of cognitive decline in mild cognitive impairment, vascular dementia, and Alzheimer disease.^14,50,51^ In these disorders, CBF is typically reduced in the posterior cingulate, precuneus, lateral parietal cortex, and medial temporal cortex in a distribution similar to the association of FRS with CBF observed here.^12,51^ Although reduced CBF is an early marker of cerebrovascular and neurodegenerative disease, CBF also gradually decreases during healthy aging.^52,53^ Importantly, FRS was associated with CBF over and above potentially confounding effects of age, GM volume, and cognitive performance.

We also observed that better cognitive performance, lower alcohol consumption, and female sex were associated with higher CBF. Because these were not our primary predictor variables and the associations were observed post hoc in a multivariable regression, they will not be further interpreted. However, this supports the extensive literature linking hypoperfusion with cognitive impairment and recent evidence linking alcohol consumption with hippocampal atrophy,^54^ so it warrants independent examination.

The differential association of FRS with CBF across phases adds to the mounting epidemiological evidence placing CVD as a midlife rather than late-life risk factor for dementia. Some methodological considerations may contribute to this age-dependent association. First, the way in which cardiovascular risk was measured may play a role. While FRS is a validated predictor of CVDs in adults aged 35 to 75 years, it may underestimate cardiovascular risk in adults older than 85 years.^21^ In our study, the mean (SD) age at the latest FRS measurement was 67.4 (4.9) years, with the oldest participant being 79.5 years old, so it is unlikely that this could have driven our results. Furthermore, attempts to adapt FRS for elderly populations have shown that neither refitting equations nor incorporating other measurements into the FRS equation improve its discriminative accuracy and that the traditionally used risk factors remained the best predictors of cardiovascular events even in the oldest participants.^55^ Second, as the FRS includes age, its age-dependent associations with CBF may be excessively driven by aging itself rather than by the other vascular risk factors. However, when we reformulated the risk score without age, our findings remained consistent. It is therefore more plausible that the age-dependent associations with CBF are associated with changes in the modifiable vascular risk factors as opposed to the nonmodifiable age component of the score. Third, as this study has a retrospective design where participants were sampled from the later phases, selection bias may have contributed to the attenuated association of FRS with CBF across phases. Participants in the Whitehall II Imaging Substudy were randomly selected from the phase 11 cohort; however, because the former required travel to Oxford, United Kingdom, and MRI compatibility, it is possible that self-exclusion may have resulted in a healthier final sample. While this may have underestimated the effect of time and particularly the association of FRS slopes with CBF, this is unlikely to be a major source of bias.

An alternative explanation is that, given the multifactorial cascade of metabolic, inflammatory, and neurodegenerative processes in aging, the unique contribution of cardiovascular risk on brain outcomes may be attenuated or even altered with age. Knopman et al^56^ found that diabetes, hypertension, obesity, and hypercholesterolemia in midlife were associated with developing mild cognitive impairment and dementia 25 years later. Similarly, Gottesman et al^57^ reported that these same vascular risk factors were associated with elevated brain amyloid deposition in midlife but not in later life. Our study suggests that the different associations of midlife and late-life vascular risk with CBF may contribute to this dichotomy, especially as dysregulation in CBF would occur upstream of β-amyloid deposition and cognitive decline. Interestingly, while midlife obesity and hypertension are associated with higher dementia risk, late-life measurements of these factors have been linked with decreased mortality.^4,5,6,7,9,58^ Therefore, it has been suggested that low body mass index and blood pressure, while protective in midlife, may be signatures of frailty and preclinical dementia in older ages.^7,9^

Overall, we found that participants’ cardiovascular health as early as their 40s may initiate processes associated with cerebral hypoperfusion in later life. Two cross-sectional studies^59,60^ have reported even earlier associations with vascular risk associated with white matter hyperintensities and poor cognitive performance in adults younger than 40 years. Taken together, this suggests that dementia prevention strategies aimed at treating cardiovascular health should start at least in young to middle adulthood; however, further studies are required to confirm this.

### Limitations

Although our models account for sex and education, the Whitehall II study sample is on average more educated and has a higher proportion of men compared with the wider population of the United Kingdom, thus limiting the generalizability of our findings. Furthermore, while we were able to tease apart midlife and late-life associations with vascular risk, we could not assess perfusion changes because pCASL scans were only acquired once. We will be acquiring longitudinal imaging in this cohort to expand on these findings in the coming years. Our study used a combined risk score and was not sufficiently powered to distinguish the contribution of individual vascular risk factors on CBF. Nonetheless, our findings may be relevant for dementia risk screenings, which assess overall cardiovascular risk profiles rather than a single risk factor alone.^61^ Our study raises questions about the mechanisms by which vascular risk can affect CBF. Future work in our group aims to examine macrovascular structure and dynamic processes of perfusion regulation, such as vascular reactivity.

## Conclusions

In this longitudinal cohort study, cardiovascular risk in midlife was significantly associated with lower GM perfusion at older ages, but this association was not significant for cardiovascular risk measured in later life. This finding could inform the timing of cardiovascular interventions so as to be optimally effective.

## Notes

Supplement. **eAppendix.** eMethods **eTable 1. **Analysis Using the Framingham Risk Score Derived Without Age **eTable 2.** Comparison of Included (n = 116) and Remaining (n = 657) Participants From the Whitehall II Imaging Substudy **eReferences**
