# Supplementary material for: Association of Midlife Cardiovascular Risk Profiles With Cerebral Perfusion at Older Ages
Source: JAMA Netw Open. 2019 Jun 21;2(6):e195776. doi: 10.1001/jamanetworkopen.2019.5776 (PMC6593638; doi:10.1001/jamanetworkopen.2019.5776)
Supplement: Supplement. — eAppendix. eMethods eTable 1. Analysis Using the Framingham Risk Score Derived Without Age eTable 2. Comparison of Included (n = 116) and Remaining (n = 657) Participants From the Whitehall II Imaging Substudy eReferences [file jamanetwopen-2-e195776-s001.pdf]

Supplementary Online Content

Suri S, Topiwala A, Chappell MA, et al. Association of midlife cardiovascular risk profiles with cerebral perfusion at older ages. *JAMA Netw Open*. 2019;2(6):e195776. doi:10.1001/jamanetworkopen.2019.5776

eAppendix. eMethods
eTable 1. Analysis Using the Framingham Risk Score Derived Without Age
eTable 2. Comparison of Included (n = 116) and Remaining (n = 657) Participants From the Whitehall II Imaging Substudy
eReferences

This supplementary material has been provided by the authors to give readers additional information about their work.

© 2019 Suri S et al. *JAMA Network Open.*

eAppendix. eMethods

Framingham CVD Risk Scores: Framingham CVD Risk Scores were derived using the standard equations[1]. As described previously[2,3], Total and HDL cholesterol (mg/dL) were measured from blood collected after either an 8-hour-fast (participants tested in the morning), or at least 4 hours after a light fat-free breakfast (participants tested in the afternoon). Cholesterol was measured using a Cobas Fara centrifugal analyzer (Roche Diagnostics System). HDL cholesterol was measured by precipitating non-HDL cholesterol with dextran sulfatemagnesium chloride and measuring cholesterol in the supernatant fluid. Systolic blood pressure (mmHg) was taken as the average of two measurements in the sitting position after 5 min rest with the Hawksley randomzero sphygmomanometer. Treated hypertension was determined according to antihypertensive medication use, including diuretics, beta-blockers, ACE-inhibitors, and calcium channel blockers. Participants were categorized as current smokers, ex-smokers, or non-smokers. Diabetes was defined by fasting glucose ≥ 7.0 mmol/L, 2 h postload glucose ≥ 11.1 mmol/L, doctor diagnosed diabetes, or use of diabetes medication.

ASL Pre-processing: The multi-PLD pCASL data were motion-corrected and registered to the calibration image using MCFLIRT. The label and control images were pair-wise subtracted and subsequently averaged using *asl_file*. The preprocessed pCASL data were used to generate perfusion maps in *oxford_asl*. Recommended fixed values for T1 of tissue (t1=1.3s), T1 of blood (t1b=1.65s) were used. The arterial transit time was estimated voxelwise from the data assuming a prior mean of 1.3 seconds, as per previous studies[4,5]. Inversion times were calculated as post-labeling delays + ATT (i.e. 1.65/1.9/2.15/2.4/2.65/2.9/3.15 s). Slice timing correction (an increase in PLD of 0.0436s per slice) and automatic spatial regularization were applied[6]. The calibration image was used to calculate the equilibrium magnetization in the ventricles, and this was converted to the equivalent value in arterial blood, accounting for differences in proton density. This was then used to give perfusion values in absolute units of ml/100g/min. For registration, a second normalised calibration image with the same resolution as the ASL data (and aligned to it) was brain-extracted and used as a reference image. T1 scans were brain-extracted using a fractional intensity threshold of 0.3 in FSL-BET. pCASL scans were registered to T1 space using a rigid registration with 6 degrees of freedom, and then to standard space using T1-to-MNI152 transformation matrices. Partial volume effects can arise from the typically low spatial resolution of pCASL data and can be a potential confounding factor given the tissue-specific kinetics (WM tends to have lower CBF and longer arrival times than GM). Partial volume effects can be particularly complicated in populations with age-related atrophy, thus an automatic partial volume correction *(--pvcorr option)* was used in BASIL[7]. This uses high-resolution partial volume estimates from the structural image to produce separate grey matter (GM) and white matter (WM) perfusion maps in both native and standard space.

© 2019 Suri S et al. *JAMA Network Open.*

eTable1. Analysis Using the Framingham Risk Score Derived Without Age

Results of regressions performed using the *Framingham CVD Risk Score*[a], and the *Modified Risk Score*[b]. All models presented below are adjusted for age, sex, education, socioeconomic and cognitive status, statins, alcohol consumption, and arterial transit time. Note that the *Modified Risk Score* has opposite signage due to the inverse transformation. Abbreviations: B(SE)=unstandardized regression coefficient (standard error), p=value for the independent variable, GM = grey matter, CBF = cerebral blood flow, CVD = cardiovascular disease, F = F statistic, df = degrees of freedom.

	**Framingham CVD Risk Score (log-transformed)** [a]	**Modified Risk Score (1/sqrt transformed)** [b]	**Interpretation**
**Results of linear regressions: B(SE), p**			
1. Change in risk score with time	0.06 (0.007), 0.000	0.04 (0.01), 0.0001	Both scores significantly changed with time
2. Total GM CBF *vs.* cumulative risk over 20 years	-0.51 (0.146), 0.0006	0.078 (0.022), 0.0005	With both scores, higher cumulative CVD risk over the 20-year period (i.e. integrals of the rate of change of risk scores) was associated with lower total GM CBF.
3. Total GM CBF *vs.* slopes and intercepts of risk trajectories	Slope: -57.79 (212.22), 0.79<br><br>Intercept: -10.50 (3.49), 0.003	Slope: 19.51 (31.65), 0.54<br><br>Intercept: 1.80 (0.48), 0.0003	With both scores, the intercepts (i.e. predicted Phase 1 risk) but not the slopes (i.e. rate of change of risk) of risk trajectories were associated with GM CBF
4. Total GM CBF vs. risk at each phase	Phase 3: -10.82 (2.87), 0.0003<br><br>Phase 5: -8.29 (2.68), 0.003<br><br>Phase 7: -8.51 (2.72), p=0.002<br><br>Phase 9: -5.74 (2.93), 0.05<br><br>Phase 11: -7.14 (2.93), 0.02	Phase 3: 1.73 (0.47), 0.0004<br><br>Phase 5: 1.26 (0.40), 0.002<br><br>Phase 7: 1.42 (0.43), 0.002<br><br>Phase 9: 0.87 (0.42), 0.04<br><br>Phase 11: 1.14 (0.42), 0.008	With both scores, Phase 3 risk was the strongest predictor of GM CBF. For the Modified Risk Score but not the Framingham Risk Score, Phase 11 risk survived the Bonferroni correction.
**Results of hierarchical regressions: F(df), p**			
5. Adding Phase 3 risk to Phase 11 model to predict GM CBF	F(1,87)=8.08, p=0.006	F(1,87)=6.62, p=0.012	With both scores, Phase 3 risk made a unique and significant contribution to GM CBF over and above the contribution of Phase 11 risk
6. Adding Phase 11 risk to Phase 3 model to predict GM CBF	F(1,87)=0.39, p=0.53	F(1,87)=0.73, p=0.40	With both scores, adding Phase 11 risk to the Phase 3 model did not significantly change model fit.

[a] At each phase, *Framingham CVD Risk Scores* were computed as described in the manuscript.
[b] At each phase, *Modified Risk Scores* were computed by removing the age component (3.06117*ln[Age] and 2.32888*ln[Age] for males and females respectively) from the Framingham CVD Risk Score equation. The resulting scores were 1/sqrt transformed due to the skewed distribution and entered into linear regressions against cerebral blood flow (CBF).

© 2019 Suri S et al. *JAMA Network Open.*

eTable 2. Comparison of Included (n=116) and Remaining (n=657) Participants From the Whitehall II Imaging Substudy

Abbreviations: FRS = Framingham cardiovascular disease risk scores.

	Included Whitehall II Imaging Sample N=116[a]		Remainder of the Whitehall II Imaging Sample N=657		Difference in means or proportions	
	N	Mean (SD) or No. (%)	N	Mean (SD) or No. (%)	Difference in means (95% CI)	p
Age, years	116	69.26 (4.96)	657	69.92 (5.23)	0.67 (-0.36 to 1.69)	0.21
Sex: female, male	116	17 (14.7%)<br>99 (85.3 %)	657	132 (20.1%), 525 (79.9%)	-0.05 (-0.02 to 0.13)	0.20
Full time education, years	116	14.05 (2.98)	657	14.43 (3.23)	-0.27 (-0.90 to 0.37)	0.41
FRS at Phase 3	116	8.22 (5.13)	588	8.02 (5.39)	-0.002 (-0.01 to 0.009)	0.71
FRS at Phase 11	116	18.84 (10.01)	624	18.31 (10.23)	-0.004 (-0.025 to 0.016)	0.67

[a]Included sample consists of the Whitehall II Imaging Sub-study participants who received an arterial spin labelling MRI scan, did not have gross structural abnormalities, and who had complete FRS data at Phase 3, Phase 11 and at least 3 Phases.

© 2019 Suri S et al. *JAMA Network Open.*

eReferences

1. D'Agostino RB, Vasan RS, Pencina MJ, et al. General cardiovascular risk profile for use in primary care: The Framingham heart study. *Circulation*. 2008;117(6):743-753. doi:10.1161/CIRCULATIONAHA.107.699579
2. Kaffashian S, Dugravot A, Brunner EJ, et al. Midlife stroke risk and cognitive decline: a 10-year follow-up of the Whitehall II cohort study. *Alzheimers Dement*. 2013;9(5):572-579. doi:10.1016/j.jalz.2012.07.001
3. Elbaz A, Shipley MJ, Nabi H, Brunner EJ, Kivimaki M, Singh-Manoux A. Trajectories of the Framingham general cardiovascular risk profile in midlife and poor motor function later in life: The Whitehall II study. *Int J Cardiol*. 2014. doi:10.1016/j.ijcard.2013.12.051
4. Okell TW, Chappell MA, Kelly ME, Jezzard P. Cerebral blood flow quantification using vessel-encoded arterial spin labeling. *J Cereb Blood Flow Metab*. 2013;33(11):1716-1724. doi:10.1002/jmri.24066
5. Harston GWJ, Okell TW, Sheerin F, et al. Quantification of serial cerebral blood flow in acute stroke using arterial spin labeling. *Stroke*. 2017. doi:10.1161/STROKEAHA.116.014707
6. Groves AR, Chappell MA, Woolrich MW. Combined spatial and non-spatial prior for inference on MRI time-series. *Neuroimage*. 2009. doi:10.1016/j.neuroimage.2008.12.027
7. Chappell MA, Groves AR, MacIntosh BJ, Donahue MJ, Jezzard P, Woolrich MW. Partial volume correction of multiple inversion time arterial spin labeling MRI data. *Magn Reson Med*. 2011. doi:10.1002/mrm.22641

© 2019 Suri S et al. *JAMA Network Open.*
